# A retrospective international study on factors associated with injury, discomfort and pain perception among cyclists

**DOI:** 10.1371/journal.pone.0211197

**Published:** 2019-01-25

**Authors:** Jose Ignacio Priego Quesada, Zachary Y. Kerr, William M. Bertucci, Felipe P. Carpes

**Affiliations:** 1 Research Group in Sports Biomechanics (GIBD), Department of Physical Education and Sports, University of Valencia, Valencia, Spain; 2 Biophysics and Medical Physics Group, Department of Physiology, University of Valencia, Valencia, Spain; 3 Department of Exercise and Sport Science, University of North Carolina at Chapel Hill, Chapel Hill, North Carolina, United States of America; 4 Laboratory of Performance, Health, Metrology, and Society (PSMS, EA 7507), UFR STAPS, University of Reims Champagne-Ardenne, Reims, France; 5 Applied Neuromechanics Research Group, Laboratory of Neuromechanics, Federal University of Pampa, Uruguaiana, Brazil; Nanyang Technological University, SINGAPORE

## Abstract

Although cycling has been associated with overuse/fatigue and acute injuries, there is lack of information regarding associated risk factors and prevention factors. The objective of the study was to determine the factors associated with injury, and perceptions of discomfort and pain in cyclists. A total of 739 cyclists completed an online questionnaire between February and October 2016. The questionnaire acquired information on participant demographics, characteristics related to cycling profile and fitness training, bike components and cycling posture, self-reported perceptions of comfort and pain, and injuries sustained in the last 12 months. Logistic regression models estimated odds ratios (OR) and 95% confidence intervals (95%CI) that examined factors associated with reporting overuse/fatigue injury, acute injury, body discomfort, saddle discomfort, and pain while cycling. Odds of reporting an overuse/fatigue injury increased when the cyclists complemented training with running (OR = 1.74; 95%CI = 1.03–2.91) or swimming (OR = 2.17; 95%CI = 1.19–3.88), and with reported pain while cycling (OR = 1.17; 95%CI = 1.05–3.69) and not cycling (OR = 1.76; 95%CI = 1.07–2.90). Odds of reporting an acute injury increased when biking to work (OR = 1.79; 95%CI = 1.07–2.86), and decreased with increased average cycling speed (1-km/h decrease OR = 0.93; 95%CI = 0.88–0.97), and compared to low-end bike, with the use of mid-range (OR = 0.25; 95%CI = 0.09–0.72) and high-end bike (OR = 0.34; 95%CI = 0.13–0.96). Although body discomfort was only associated with saddle discomfort and the presence of pain during cycling, saddle discomfort was also associated with biking to work (OR = 0.46; 95%CI = 0.22–0.88). Finally, pain perception was associated with a number of factors such as ride to work, core training, cycling experience, saddle discomfort, pain while not cycling. Numerous factors are associated with injury, and perceptions of discomfort and pain in cyclists. Such factors should be considered when developing training routines, bicycle maintenance best practices, and injury prevention programs.

## Introduction

Cycling is a popular sport worldwide [1,2] that has been found to not only improve fitness and health, but also assist rehabilitation regimes due to its lower impact on the joints in comparison to other activities such as walking and running [3,4]. Despite these benefits, cycling may also result in overuse/fatigue and acute injuries [2,5,6]. Decock et al. (2016) examined cycling competitions in 2012 and found that 15.8% of the cyclists sustained an injury. Barrios et al. (2015) found that 54% and 46% of injuries among professional cyclists are related to acute and overuse injuries, respectively.

While previous research has reported the frequency and type of cycling-related cycling [2,5–7], there less research related to the associated risk factors and prevention factors, both of which are fundamental the development of prevention programs that aim to reduce the incidence and severity of injuries [8]. Although studies have hypothesized that risk factors for cycling injuries include sex [2,9] and age [2], modifiable factors such as cycling profile or bicycle equipment [10–12] are also worth examining.

As observed in other sports, perceived discomfort and pain during physical activity may be early indicators of injury risk [13–15]. Furthermore, these perception variables are important in the context of cycling because cycling practices that are painful and uncomfortable could result in reduced performance and even result in the abandonment of the activity. Perceived comfort during cycling can depend on bike components, cycling posture, and environmental factors (e.g. road conditions) [12,16]. Likewise, perceived pain may be associated by certain factors, such as the type of physical activities in which athletes participate [17]. For cycling, perceived pain may be useful in understanding factors associated with comfort.

More in-depth examinations of injury risk and perceived pain and discomfort are warranted. As a result, our study examined factors associated with overuse/fatigue and acute injury, perceived discomfort, and perceived pain in a sample of cyclists. This sample was acquired through the worldwide dissemination of a questionnaire created in multiple languages.

## Materials and methods

### Design

Our study was cross-sectional in design and followed STROBE guidelines [18]. A self-administered questionnaire was made accessible online worldwide from February 2016 to October 2016. The study complied with the Declaration of Helsinki and was approved by the ethics committee from the Universitat de València (approval number H1449762164108). Participants approved the informed consent in the first page of the online questionnaire.

### Participants

The questionnaire was sent to several cycling organizations, cycling clubs, Internet blogs, associations, and posted on several internet forums intended for cyclists. These organizations and websites were based in numerous countries, with variations as to whether their audience was national or global.

To be eligible for this study, the respondent had to be: literate in English, Spanish, French, or Portuguese; aged 18 years or older; and be involved in non-sporadic cycling (≥2 rides/week, ≥50 km/week and ≥3 hours of cycling/week). We excluded those respondents submitting data that were incomplete or containing questionable values (e.g., body mass reported as 5 kg). Cycling modalities with less than 50 respondents were also excluded (e.g., BMX modality).

### Questionnaire

Table 1 highlights the variables captured by the survey as well as the response options for each variable. To improve the comprehension of some of the questions, images were provided. Although we made a priori decisions on how to recode certain variables for analysis, such recoding was further modified in those cases in which resulting category counts were low in number. In this study, injury was defined as any physical complaint sustained by the participant, irrespective of the need for medical attention or time loss from cycling activities [19,20].

**Table 1:** Information requested on the questionnaire.

Group of items	Name variable	Description item	Response options	Recoding of data by authors
Demographics	Gender	Gender of the participant.	Male; Female	-
	Age	Age in years.	Open field	-
	Height	Height of the participant.	Open field	Height and body mass reported in feet/inches and pounds were converted to cm and kg, respectively.
	Body Mass	Body mass of the participant.	Open field	
	BMI	Body Mass Index.	-	BMI was determined using the self-reported height and body mass.
	Country	Current country of residence of the participant.	Open field	Country was recoded as Spain, France, Brazil, and others.
	Continent	Current continent of residence of the participant.	Africa; Asia; Europe: North America; South America; Antarctica; Australia	Continent was recoded as Europe, South America, and others.
	Race/ethnicity	Race/ethnicity of the participant.	White/Caucasian; Middle Eastern; Black/African; American Indian/Alaska Native; Asian/Indian; Latino/Hispanic; South American; Other	-
Characteristics of cycling profile within the last 12 months	Bike to work	If they use a bicycle to commute to work.	Yes; No	-
	Cycling frequency	Frequency of cycling in rides per week.	Open field	-
	Volume hours	Weekly volume of cycling in hours.	Open field	-
	Volume km	Mileage of cycling per week.	Open field	-
	Total volume km	Total weekly volume of cycling using also the km to commute to work.	Open field	-
	Experience	Cycling experience in years of training.	Open field	-
	Speed	Average cycling speed during training sessions in km/h.	Open field	-
	Modality	Type of cycling modality.	Road; Mountain bike; Triathlon; BMX; Open field	-
	Purpose	Cycling purpose.	Professional competition; Recreational competition; Recreational without competition	-
	Terrain	Terrain more often faced during cycling.	Road; Trail; Open field	-
	Coach	If they have professional support (e.g. coach) for their cycling training.	Yes; No	-
	Smartphone	If they use a smartphone application to assist cycling training regime/schedule.	Yes; No	-
Characteristics of fitness training within the last 12 months	Core training	If they complement cycling with core training.	Yes; No	-
	Flexibility training	If they complement cycling with flexibility training.	Yes; No	-
	Strength training	If they complement cycling with strength training/weight lifting.	Yes; No	-
	Sport	If they complement cycling with other sport or training.	Yes and which (open field); No	Sports listed were running, swimming, team sport, racquet sport, gym sport, and walking sport (for each variable the answers were yes or no).
Bike characteristics and cycling posture about the most used bike	Bikes owned	Number of bicycles owned.	Open field	Bikes was recoded as 1 and >1.
	Size	If when they bought their bicycle receive instructions regarding selecting the correct size.	Yes; No	-
	Maintenance	If when they bought their bicycle receive instructions regarding maintenance.	Yes; No	-
	Quality	Which is the quality that they consider that have their bike.	Low-end; Mid-range; High-end	-
	Suspension	If the bike has a suspension system.	Front suspension; Rear suspension; Full suspension; No	-
	Chain-ring	Kind of chain-ring. This item was supported with an image.	Circular; Non-circular; IDK	-
	Objective posture	Most important aspect for them regarding their cycling position.	Maximum performance; Maximum comfort; Balance between both	-
	Crank arm	Size of crank arm.	170; 172.5; 175; Open field; IDK	Crank arm was recoded as correct, not correct and IDK. For this recodification, because the inseam length is very correlated with the height and it is considered the 45% of the height [21–23], inseam length was calculated and compared with the suggested proposal of crank assignation of Geoff Drake [24], where 165 is appropriate for inseams length <73.5 cm, 170 for inseams lengths between 73.5–81.5 cm, 172.5 for inseam lengths between 81.5 and 86.5 cm, and 175 for inseams lengths >86.5 cm.
	Cycling shoes	If they wear cycling shoes.	Yes; No	-
	Cleats	How they adjust their cleats.	Adjusted by respondent; Adjusted by professional; Not adjusted; Not use cleats	-
	Aerobars	If they use aerobars. This item was supported with an image.	Yes; No	-
	Body comfort	Classification of their body comfort during cycling.	Very comfortable; Comfortable; Uncomfortable; Very uncomfortable	Body comfort and Saddle comfort were recoded as discomfort reported (yes/no) for statistical models
	Saddle comfort	Classification of their saddle comfort during cycling.	Very comfortable; Comfortable; Uncomfortable; Very uncomfortable	
Pain	Pain during cycling	If they experience pain during cycling practice and in which body areas.	No; Neck; Shoulder; Upper back; Arm; Hand; Lower back; Hip; Genital area; Anterior thigh; Posterior thigh; Knee; Leg; Ankle; Foot	Pain during cycling and pain while not cycling were recoded as reported pain (yes/no) for the statistical models. The pain areas were analyzed separately.
	Pain while not cycling	If they experience pain while not cycling and in which body areas.	Same responses as Pain during practice	
Injuries in the last 12 months. If the participant had more than 4 injuries in the last 12 months, they were instructed to provide information about the most recent 4.	Injuries	Number of injuries.	0; 1; 2; 3; 4 and more	Number of injuries was recoded for statistical models as injured/not injured
	Region injury	Body region of each injury.	Same regions as Pain practice	-
	Diagnosis injury	Diagnosis of each injury.	Sprain/strain; Contusion/abrasion; Concussion; Fracture/stress fracture; Inflammatory conditions; Muscle ruptures and micro-ruptures; Laceration; Other (open field)	The category of “degenerative injuries” was included in diagnosis after review the responses of participants.
	Cause injury	Perception of the cause of each injury.	Fall; Contact with vehicle; Contact with other bicyclist; Contact with pedestrian; Contact with stand-still Structure; Incorrect posture; Incorrect pedaling technique; Overuse/fatigue; Playing another sport; Unknown; Other (open field)	-
	Medical leave	If the injury produced a medical leave.	Yes; No	-
	Surgery	If the injury required a surgical intervention.	Yes; No	-
	Recovery	The duration of recovery time for each injury.	<1 day; 1 day to <1 week; 1 week to <2 weeks; 2 weeks to <1 month; 1 month to <3 months; ≥3 months	-

IDK: I don´t know.

To obtain a large and diverse sample of cyclists, we created the questionnaire in four languages (English, Spanish, French, and Portuguese). All four authors assisted in the translations to ensure consistency across the versions. The survey was hosted in Google Forms.

### Statistical analysis

Statistical analyses were performed using RStudio [25]. Descriptive analyses were performed to acquire averages and 95% confidence intervals (95%CI). Logistic regression analyses were performed to estimate odds ratios (OR) and 95%CI. A total of seven models were run. Outcome variables of interest were: reporting any injury within the last 12 months (recoded as injured within the last 12 months: yes/no); reporting an overuse/fatigue injury within the last 12 months (injuries in which participants perceived overuse/fatigue as the cause); reporting an acute injury within the last 12 months (injuries that participants perceived that the cause was fall, contact with vehicle, other bicyclist, pedestrian or with stand-still structure); reporting body comfort (recoded as body discomfort reported: yes/no); reporting saddle comfort (recoded as saddle discomfort reported: yes/no); reporting pain during cycling (recoded as yes/no); and reporting pain while not cycling (recoded as yes/no). The exposure variables included the variables captured from: demographics; characteristics of cycling profile within the last 12 months; characteristics of fitness training within the last 12 months; and bike characteristics. Stepwise multiple regressions in both directions were performed to find the model with the best AIC (Akaike Information Criterion) [26]. Final models were then adjusted to retain only variables yielding p-values <0.05.

## Results

**Fig 1:**
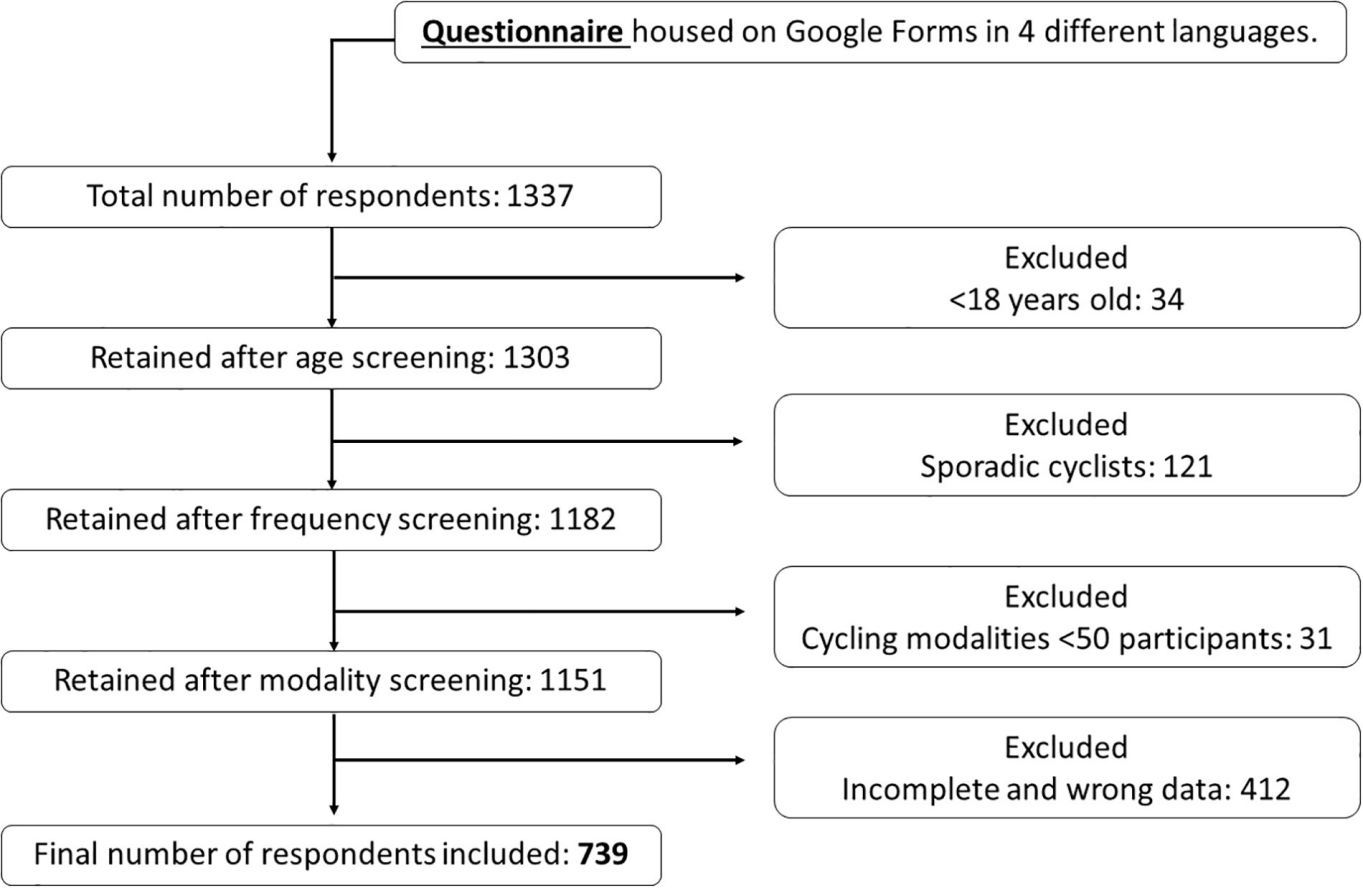
Acquisition of final sample size used for analyses. Sporadic participants were considered those respondents reporting <2 ride/week, <50 km/week, and/or <3 hour of cycling/week.

Of the 1337 respondents, the final dataset for analyses included 739 cyclists. Fig 1 illustrates the flowchart that highlights the reasons for exclusion from analyses. The final sample included 677 males and 62 females, with an average age of 39.3 ±10.8 years and an average body mass index (BMI) of 24.1 ±2.8. Most respondents were from Europe (n = 460), followed by South America (n = 235). On average, respondents cycled 3.6 ±1.3 days per week, rode 203.2 ±110.0 km per week, and had 12.6 ±10.3 years of cycling experience. Most of respondents were road cyclists (n = 450), followed by mountain bike cyclists (n = 234), and triathlon cyclists (n = 55).

### Injuries reported in last 12 months

Most cyclists reported no injuries in the past 12 months (63.2%; 95%CI [59.7–66.7%]), 25.3% reported one injury (95%CI [22.2–28.5%]), and 11.5% reported two or more injuries (95%CI [9.2–13.8%]). Injury characteristics are presented in the Table 2.

**Table 2:** Characteristics of injuries reported by participants within the last 12 months. Percentages were obtained from the total number of cyclists injured (n = 275).

	N	%	95% CI
**Body region injured**			
Knee	82	30.2	24.7−35.6
Lower back	51	18.8	14.1−23.4
Shoulder	40	14.7	10.5−18.9
Hip	28	10.3	6.7−13.9
Hand	26	9.6	6.0−13.1
Leg	23	8.5	5.1−11.8
Ankle	23	8.5	5.1−11.8
Elbow	18	6.6	3.6−9.6
Thigh	16	5.9	3.1−8.7
Neck	12	4.4	2.0−6.9
Upper back	11	4.0	1.7−6.4
Arm	6	2.2	0.5−4.0
Chest	4	1.5	0.0−2.9
Head	3	1.1	0.0−2.4
**Diagnosis**			
Inflammatory conditions	120	44.1	38.2−50.1
Sprain/strain	66	24.3	19.1−29.4
Contusion/abrasion	43	15.8	11.5−20.2
Fracture/stress fracture	36	13.2	9.2−17.3
Muscle ruptures and micro-ruptures	17	6.3	3.4−9.1
Laceration	7	2.6	0.7−4.5
Degenerative	6	2.2	0.5−4.0
Concussion	1	0.4	0.0−1.1
Other	20	7.4	4.2−10.5
**Perceived main cause of the injury**			
Overuse/fatigue	89	32.7	27.1−38.3
Fall	84	30.9	25.4−36.4
Unknown	41	15.1	10.8−19.4
Incorrect posture	30	11.0	7.3−14.8
During playing another sport	21	7.7	4.5−10.9
Incorrect pedalling technique	11	4.0	1.7−6.4
Contact with stand-still structure	9	3.3	1.2−5.5
Contact with other bicyclist	7	2.6	0.7−4.5
Contact with vehicle	6	2.2	0.5−4.0
Contact with pedestrian	2	0.7	0.0−1.8
Other	17	6.3	3.4−9.1
**Medical leave**			
Yes	79	29.0	23.6−34.5
**Recovery time**			
<1 day	7	2.6	0.7−4.5
1 day to <1 week	38	14.0	9.8−18.1
1 week to <2 weeks	48	17.7	13.1−22.0
2 weeks to <1 month	91	33.5	27.8−39.1
1 month to <3 months	81	29.8	24.3−35.3
>3 months	58	21.3	16.4−26.2

Note: inflammatory conditions included but were not limited to: bursitis, tendonitis, and other unspecified inflammation.

Table 3 presents the logistic regression models for being injured in the past 12 months. The odds of reporting an injury within the past 12 months increased when cyclists also engaged in running, rode on trail terrain (compared to road terrain), biked to work, and reported pain during cycling and while not cycling. The odds of reporting an overuse/fatigue injury increased when the cyclist was also engaged in running and swimming, and reported pain during cycling and while not cycling. The odds of reporting an acute injury increased when cyclists biked to work, rode at lower speed, and had a low-end bike (compared to mid-end and high-end bikes).

**Table 3:** Logistic regression model to assess the odds of reporting any injury, overuse/fatigue injury, or acute injury within the past 12 months. Referent category was not reporting an injury.

**Model Predicting Odds of Injury within the past 12 months**		
**Predictor variable**	**Odds Ratio**	**95%CI**
Engaged in running		
Yes^(ref.no)^	1.76***	1.27, 2.45
Terrain type while cycling		
Trail^(ref.road)^	1.46**	1.02, 2.10
Bike to work		
Yes^(ref.no)^	1.63**	1.16, 2.30
Pain during cycling		
Yes^(ref.no)^	1.53*	1.08, 2.18
Pain while not cycling		
Yes^(ref.no)^	1.78***	1.28, 2.48
**Model Predicting Odds of Overuse/Fatigue Injury within the past 12 months**		
**Predictor variable**	**Odds Ratio**	**95%CI**
Engaged in running		
Yes^(ref.no)^	1.74*	1.03, 2.91
Engaged in swimming		
Yes^(ref.no)^	2.17*	1.19, 3.88
Pain during cycling		
Yes^(ref.no)^	1.17*	1.05, 3.69
Pain while not cycling		
Yes^(ref.no)^	1.76*	1.07, 2.90
**Model Predicting Odds of Acute Injury within the past 12 months**		
**Predictor variable**	**Odds Ratio**	**95%CI**
Bike to work		
Yes^(ref.no)^	1.76*	1.07, 2.86
Speed (per 1-km/h increase)	0.93***	0.88, 0.97
Bike quality		
Mid-range^(ref.Low-end)^	0.25**	0.09, 0.72
High-end^(ref.Low-end)^	0.34*	0.13, 0.96

***p<0.001;

**p<0.01;

*p<0.05

### Comfort

Most cyclists reported their body posture during cycling to be comfortable (95.8%; 95%CI [94.4–97.3%]); 4.2% reported discomfort (95%CI [2.8–6.0%]). Most cyclists reported their saddle to be comfortable (89.3%; 95%CI [87.1–91.5%]); 10.7% reported discomfort with their saddle (95%CI [8.5–12.9%]).

Table 4 presents the logistic regression models predicting the odds of self-reported body posture discomfort and saddle discomfort. The odds of reported body posture discomfort increased with reporting saddle discomfort and pain while cycling. The odds of self-reported saddle discomfort increased with reporting body posture discomfort and perceived pain during cycling, but decreased when cyclists reporting biking to work.

**Table 4:** Logistic regression models assessing the odds of reporting body posture discomfort and saddle discomfort while cycling, respectively. Referent categories for models were not reporting body discomfort and saddle discomfort while cycling, respectively.

Model Predicting Odds of Body Posture Discomfort			Model Predicting Odds of Saddle Discomfort		
Predictor variable	Odds ratio	95%CI	Predictor variable	Odds ratio	95%CI
Saddle discomfort			Bike to work		
Yes^(ref.no.)^	9.49***	4.68, 19.73	Yes^(ref.no)^	0.46*	0.22, 0.88
Pain during cycling			Body posture discomfort		
Yes^(ref.no)^	5.67*	1.63, 35.72	Yes^(ref.no.)^	9.35***	4.31, 20.70
			Pain during cycling		
			Yes^(ref.no)^	5.36***	2.56, 13.08

***p<0.001;

**p<0.01;

*p<0.05

### Pain

While 36.8% of cyclists did not report perceived pain while cycling (95%CI [33.3–40.3%]), 63.2% reported perceived pain (95%CI [59.7–66.7%]). The body regions where pain was most often reported were: neck (23.1%; 95%CI [20.1–26.2%]), lower back (22.2%; 95%CI [19.2–25.2%]), knee (15.6%; 95%CI [12.9–18.2%]), hand (13.3%; 95%CI [10.8–15.7%]), genital area (11.6%; 95%CI [9.3–14.0%]), and shoulder (11.1%; 95%CI [8.8–13.4%]).

Overall, 58.2% of cyclists noted no pain when they were not cycling (95%CI [54.6–61.8%]); 42.0% reported pain while not cycling (95%CI [38.3–45.4%]). The body regions where pain was most often perceived were: lower back (19.4%; 95%CI [16.5–22.2%]), knee (11.6%; 95%CI [9.3–14.0%]), and neck (8.8%; 95%CI [6.8–10.8%]).

Table 5 presents the logistic regression models predicting the odds of reporting perceived pain during cycling and while not cycling. The odds of reporting perceived pain during cycling increased with reporting saddle discomfort, perceived pain while not cycling, and injury within the past 12 months; however, biking to work, having more experience, and training the core musculature were associated with decreased odds for perceived pain. The odds of reporting perceived pain while not cycling increased with reporting perceived pain while cycling and injury within the past 12 months; however, odds decreased when also engaged in running. In addition, the odds of reporting perceived pain while not cycling also varied by cleats use/adjustment.

**Table 5:** Logistic regression models assessing the odds of reporting pain while cycling and pain while not cycling, respectively. Referent categories for models were not reporting pain.

Model Predicting Odds of Pain While Cycling			Model Predicting Odds of Pain While Not Cycling		
Predictor variable	Odds Ratio	95%CI	Predictor variable	Odds Ratio	95%CI
Bike to work			Engaged in running		
Yes^(ref.no)^	0.60**	0.41, 0.87	Yes^(ref.no)^	0.69*	0.50, 0.95
Experience (per 1-year increase)	0.98**	0.96, 0.99			
Training core musculature			Cleats		
Yes^(ref.no)^	0.68*	0.49, 0.95	Adjusted by cyclist^(ref.not use)^	1.37	0.64, 3.02
Saddle discomfort			Adjusted by professional^(ref.not use)^	1.46	0.67, 3.28
Yes^(ref.no)^	5.78***	2.71, 14.31	Not adjusted^(ref.not use)^	4.56**	1.53, 14.30
Pain while not cycling			Injury within past 12 months		
Yes^(ref.no)^	5.02***	3.49, 7.30	Yes^(ref.no)^	1.84***	1.36, 2.49
Injury within past 12 months					
Yes^(ref.no)^	1.46*	1.03, 2.10			

***p<0.001;

**p<0.01;

*p<0.05

## Discussion

Previous research has examined risk factors associated with cycling [2,9]. However, examinations of reported discomfort and perceived pain while engaged in an activity is just as important, particularly as previous research in other sports have found such factors to be early indicators of injury risk [13–15]. Alongside the continued research to examine factors associated with injury in cyclists, we also examined factors associated with discomfort and perceived pain while cycling and while not cycling (i.e. off the bike during the daily activities). In our sample of 743 cyclists, we found that reporting injury, discomfort, and perceived pain to be associated with: cycling profiles (terrain, if they use the bike to commute to work, average speed, and cycling experience); characteristics of fitness training (if they complement cycling with running, swimming, or core training); and bicycle equipment (bike quality and cleats use/adjustment). Such findings may be of benefit to the cycling communities to help identify prevention programming to reduce the incidence of injury, as well as the clinicians that provide injury prevention and care to this population. Furthermore, such information may also help to ensure that as encouragement of physical activity through cycling is promoted, so is the prevention of injury.

### Factors associated with cycling-related injury and resulting prevention

As found in previous research, the knee, lower back, and shoulder were commonly injured body regions among cyclists [5,6,27,28]. Common diagnoses include inflammatory conditions, sprain/strain, and contusion [5,28,29]. Fatigue and falls are the main causes for these injuries [28,30,31]. The need for continued examination of strategies to prevent cycling-related injury is highlighted by our finding that 28.7% of injured respondents required medical leave; most injuries resulted in recovery time durations of over 2 weeks. Thus, effective prevention strategies may benefit from focusing on these common injury types. In additional, clinical research should examine management and care guidelines that can help cyclists safely return to participation and avoid risk of further complications from injury.

Reporting overuse/fatigue injury within the past 12 months was associated with engaging in running or swimming training. This finding may be plausible since it has been found that cyclists who combine more than one discipline tend to have poor technique, which has been suggested with increased risk of injury [32]. Further, previous studies have found that triathletes produce less effective force and have a greater variation in muscle recruitment than cyclists [33,34]. Additionally, combining running, swimming and cycling without a proper periodization of the training can lead to overload and fatigue, both of which are previously-identified risk factors (also were the main perceived reason of injury by cyclists in our study) [32,35]. However, as a cross-sectional study, we are unable to assess causality. It is possible that running or swimming training may have also been undertaken while recovering from the cycling injury. Future research is needed to further examine the relationship between engaging in multiple forms of physical activity and an increased injury risk.

Compared to road cycling, cycling on trail terrain increased the odds of injury. It is important to note that our terrain categories could not account for variations within each terrain, such as changes in traffic presence in road and trail courses. However, our findings may be attributable to the fact that whereas road cycling has a more level terrain, trail terrain is more varied and thus, results in more vibrations on the bicycle and the cyclist. Previous research has suggested that high levels of vibration on the bicycle may be associated with musculoskeletal disorders, particularly related to the upper extremity, the knee, and the back [36,37]. This additional mechanical load could also increase muscular activity, with such articular strain contributing to injury risk [36,37]. Cyclists engaged in trail terrain cycling may benefit from the use of recovery strategies such as cryotherapy, stretching compression garments, nutrition, or massage therapy under medical supervision [38,39].

Cycling to work was associated with increased odds of reporting an acute injury in the past 12 months. This result may highlight the need for safety-related infrastructure for cyclists as they commute [40,41]. The inverse relationship found between the cyclist’s average ride speed and the probability of having an acute injury may reflect the greater acute injury risk within a city setting, where cycling speed is typically slower. Last, decreased odds of acute injury were found when riding a high-end or mid-range quality bike, compared to a low-end quality bike. Although not measured specifically in our study, such results may highlight the higher quality of components on these bikes (e.g., brakes). Future research is needed to better understand the specific mechanisms by which these associations were found.

### Factors associated with reported discomfort and pain

Building upon etiological research focused on injury incidence in cycling, our study also aimed to identify factors associated with perceived discomfort and pain. We hypothesized that discomfort and pain related to cycling may be associated with injury risk. Although our logistic models only found an association between perceived pain and injury, we believe continued exploration of these associations is required with additional samples from the cycling population to further validate or refute our findings. In addition, we believe that examining perceived discomfort and pain are nonetheless important as they may provide valuable information to drive the development of cycling-related injury prevention strategies. Such findings may be of interest to the many stakeholders within the cycling population, including coaches, bike fitting technicians, and medical staff treating such injuries.

Different studies observed how the modification of saddle discomfort was related with factors such as the variability of the sitting postural control [42,43], trunk flexion [44], forward-backward sitting position, and neuromuscular activation of gastrocnemius [43]. A previous study observed that saddle discomfort increases with cycling time [43]. Riding duration can explain why in our study the cyclists that bike to work presented lower odds of saddle discomfort. Although discomfort increases with cycling time [43], it is unknown how this discomfort can alter cycling posture, neuromuscular activation, or pedal forces, thus warranting future examination. In our study, approximately 1 in 10 of the cyclists reported saddle discomfort. Saddle discomfort should not be ignored since this can be the result of a high compression in the gluteal area that is often accompanied by skin discomfort, syndromes such as urinary pain, and numbness in the perineal region [45–47].

Interestingly, we found that the odds of reporting pain while cycling decreased with more cycling experience and when the cyclist reported cycling to work. It is likely that respondents with a higher exposure to cycling may have acclimated themselves to a higher pain threshold [29,48]. This is in agreement with previous studies that found that aerobic exercise was associated with higher pain tolerance [49,50]. The increase in pain tolerance in cycling is important because it was suggested to be an important factor for endurance performance [48]. Also, cyclists that trained their core musculature had decreased odds of reporting pain while cycling. This parallels previous findings suggesting that lumbar pain was reduced by lumbopelvic stabilization training [51]; more specifically in cycling, such pain was also associated with lower control of the trunk musculature [52,53]. Finally, cyclists without adjustments made to their cleats had increased odds of reporting pain while not cycling. A wrong adjustment of the cleats can lead to an excessive Q-angle, an important factor associated with chondromalacia [54]. It is important to note that we measured perceived pain through self-report, which may result in limitations. However, our findings highlight the need to further examine perceived pain in a variety of manners in the context of injury risk to better gauge how training load, pain threshold, and willingness to participate while in pain are related.

### Limitations

As in other studies [5,27], a prospective design and a data collection by interviews was considered. Although the use of web-based questionnaires has been supported by the literature [55,56], it is important to be aware of their limitations. One limitation is combining data from different methods of survey, which was avoided here by using the same instrument for all participants, being the language the only difference [57]. The range of languages used and the countries targeted must be considered. Although we included four different languages, it is possible that participation of some countries could be higher if providing the questionnaire in additional languages. Definitions for each injury diagnosis were not included in the questionnaire, which could have potentially lead to respondents incorrectly stating their respective injury diagnoses. Finally, despite the large sample size, we had a larger proportion of respondents that were males than females. Future research focused on females and the potential risk factors associated with cycling is warranted.

## Conclusion

Our findings highlight the many factors associated with cycling-related injury, and perceived discomfort and pain. Furthermore, these findings may contribute to the development of prevention strategies that will help decrease the incidence of cycling-related injury, while also considering factors related to perceived discomfort and pain while cycling. It is important for clinicians working with cyclists to understand such associated risk and preventive factors to help guide recommendations for injury prevention, care, and management.
